# Bilobar Radioembolization Carries the Risk of Radioembolization-Induced Liver Disease in the Treatment of Advanced Hepatocellular Carcinoma: Safety and Efficacy Comparison to Systemic Therapy with Atezolizumab/Bevacizumab

**DOI:** 10.3390/cancers15174274

**Published:** 2023-08-26

**Authors:** Matthias Jeschke, Johannes M. Ludwig, Catherine Leyh, Kim M. Pabst, Manuel Weber, Jens M. Theysohn, Christian M. Lange, Ken Herrmann, Hartmut H. -J. Schmidt, Leonie S. Jochheim

**Affiliations:** 1 Department of Gastroenterology, Hepatology and Transplant Medicine, University Hospital Essen, University of Duisburg-Essen, 45147 Essen, Germany; 2 Institute of Diagnostic and Interventional Radiology and Neuroradiology, University Hospital Essen, University of Duisburg-Essen, 45147 Essen, Germany; 3 Clinic for Gastroenterology, Hepatology and Infectious Diseases, Medical Faculty of Heinrich Heine University Düsseldorf, University Hospital Düsseldorf, 40225 Düsseldorf, Germany; 4 Department of Nuclear Medicine, University Hospital Essen, University of Duisburg-Essen, 45147 Essen, Germany; 5 German Cancer Consortium (DKTK), University Hospital Essen, 45147 Essen, Germany

**Keywords:** hepatocellular carcinoma, atezolizumab, bevacizumab, immune checkpoint inhibitors, systemic treatment, radioembolization, hepatotoxicity, Yttrium-90, ALBI, REILD

## Abstract

**Simple Summary:**

Treatment options for intermediate- or advanced-stage hepatocellular carcinoma include trans-arterial radioembolization of the whole liver and systemic therapy. While no significant difference in overall survival has been reported for patients receiving radioembolization or sorafenib, in this study, we compared radioembolization and treatment with atezolizumab/bevacizumab, which has recently become the de facto first-line treatment option in advanced stage unresectable HCC. Overall survival in the TARE group was limited by the risk of radioembolization-induced liver disease (REILD). Baseline liver function was a predictor for the occurrence of REILD.

**Abstract:**

Recommended treatment options for advanced-stage hepatocellular carcinoma (HCC) include systemic therapy (ST) and trans-arterial radioembolization (TARE) with Yttrium-90 (Y90). Before the approval of immune-checkpoint inhibitors, a similar safety profile was reported for TARE and ST with tyrosine kinase inhibitors (TKI). However, whole-liver treatment and underlying cirrhosis were identified as risk factors for potentially lethal radioembolization-induced liver disease (REILD). Therefore, the safety and efficacy of TARE and ST with atezolizumab/bevacizumab were compared in patients with advanced HCC involving at least both liver lobes in a retrospective real-world cohort. In total, 74 patients with new or recurrent advanced-stage HCC (BCLC stage B/C) were included if treated with either bilobar TARE (n = 33) or systemic combination therapy with atezolizumab plus bevacizumab (n = 41). Most patients had compensated liver function (90.5% were classified as Child-Pugh Score A, 73% as ALBI Grade 1) at baseline. Although not significant, patients treated with ST showed a more prolonged overall survival than those treated with Y90 TARE (7.1 months vs. 13.0 months, *p* = 0.07). While a similar disease control rate could be achieved with bilobar TARE and atezolizumab/bevacizumab, in the TARE group, overall survival was curtailed by the occurrence of REILD. In patients with underlying liver cirrhosis, the liver function at baseline was a predictor for REILD.

## 1. Introduction

Hepatocellular carcinoma (HCC), the most common form of primary liver cancer, remains a global health burden. As the sixth most common cancer worldwide, it accounted for the third most cancer-related deaths in 2020 [1]. Despite implementing HCC screening programs for patients at risk, diagnosis often occurs in advanced stages, in which patients are ineligible for curative treatment options such as surgical resection, ablation, or transplantation.

Over the past two decades, transarterial radioembolization (TARE) has emerged as an effective treatment for locally advanced HCC at stages BCLC B/C [2], providing a viable alternative to systemic therapies (ST). TARE, also referred to as selective internal radiotherapy (SIRT), is a form of brachytherapy wherein yttrium-90 microspheres, serving as the radiation source, are delivered through the hepatic artery or its branches. According to the current BCLC treatment algorithm, TARE is recommended for the treatment of HCC in early stages, especially for unifocal lesions measuring below 8 cm when other options such as transarterial chemoembolization (TACE), ablation, transplantation, or resection are not feasible [3]. In this stage, patients are classically considered for treatment with TACE, in which cytotoxicity and ischemia are induced in the tumor by intra-arterial infusion of chemotherapy, followed by embolization of the feeding vessels [4,5]. However, recent studies indicate that Y90 treatment surpasses TACE efficacy, as demonstrated, for example, in the TRACE II trial conducted by Dhondt et al. [6,7].

Before the approval of immune checkpoint inhibitors (ICI), then-current guidelines suggested TARE as an alternative treatment also in patients with advanced HCC (stage BCLC C) when liver function was preserved [2,5]. It was argued that systemic therapies have the potential to induce toxic effects while possibly lacking a significant treatment benefit. In contrast, TARE usually has a direct and observable effect on the tumor, boasting response rates of up to 50% and disease control rates above 90% [8,9,10] with only mild toxicity reported. However, two large randomized controlled trials comparing the efficacy and safety of treatment with TARE and Sorafenib could not demonstrate a more prolonged overall survival for patients in the TARE group [11,12]. Although fewer serious adverse events were reported for the TARE groups in both studies, treatment-related toxicity remains a concern.

Among others, radioembolization-induced liver disease (REILD)—hepatic decompensation due to radiation toxicity—constitutes a potentially lethal complication. The reported incidence of REILD varies greatly between 2.2% and 31% [13] but is commonly associated with underlying liver cirrhosis or prior exposition to toxicity due to chemotherapy or external beam radiation [14]. Another risk factor for the occurrence of REILD is whole-liver treatment since no non-tumorous liver parenchyma is spared [15].

Recent years have also seen significant changes in the systemic treatment landscape for HCC. Following the results of the pivotal IMbrave150 trial, the combination of the anti-programmed death ligand-1 (PD-L1) inhibitor atezolizumab and the vascular endothelial growth factor (VEGF) inhibitor bevacizumab has become the new standard of care for first-line treatment of patients with advanced unresectable hepatocellular carcinoma (BCLC B/C). Compared to standard therapies such as sorafenib, ICIs have also shown favorable adverse event profiles, with grade 3 and 4 treatment-related adverse events occurring in only 18 to 22% of cases [16]. However, to the authors’ knowledge, no head-to-head comparison between TARE and atezolizumab/bevacizumab has yet been performed.

Given these latest developments in the systemic treatment landscape, we aimed to compare the efficacy and safety of whole-liver TARE with systemic therapy for the treatment of advanced HCC in a real-world cohort and identify patients at risk for developing REILD.

## 2. Materials and Methods

The University Hospital Essen (Essen, Germany) ethics committee approved the study, which was conducted per the 1964 Declaration of Helsinki and its later amendments. Written informed consent was obtained from all patients.

### 2.1. Study Design and Patients

In this retrospective study, we included all consecutive adult patients with HCC, who were allocated to treatment with either whole-liver radioembolization or systemic combination therapy with atezolizumab and bevacizumab between January 2019 and October 2021 at the University Hospital Essen by a multidisciplinary oncological team. TARE or ST was recommended in patients not amenable to other treatments. Decisions were based on the BCLC treatment algorithm and ESMO guidelines, as well as individual oncological history and patient preferences. Generally, TARE was only performed in patients with preserved liver function (Child-Pugh Score (CPS) ≤ 7), a tumor burden of less than 50%, and when there was no clear evidence of extrahepatic spread at baseline except for possibly portal vein invasion. Patients with fibrolamellar HCC, mixed hepatocellular cholangiocarcinoma, or who had undergone previous whole-liver TARE or ST were excluded.

### 2.2. Definitions

REILD was defined according to Braat et al. [13] as hepatic decompensation developing after TARE in the absence of tumor progression or biliary obstruction. Specifically, we defined hepatic decompensation in this context as an increase in CPS of at least 2, with most patients developing jaundice or new-onset ascites. While the histopathological hallmark of REILD is a veno-occlusive disease, a liver biopsy was not collected in our patients developing REILD to confirm the diagnosis, as they most often had ascites and a diminishing general condition (performance status ≥ ECOG 3), where therapy shifted towards best supportive care. Regarding the time criterion—REILD is reported to develop two weeks to four months after treatment—it was met in all but one of our patients, who showed hepatic decompensation on the day following the second TARE procedure.

### 2.3. Treatments

TARE with Y-90 microspheres (TheraSphere, Boston Scientific, Marlborough, MA, USA) was performed as described in detail elsewhere [17]. In summary, patients were studied before treatment by an angiogram to identify vessels providing arterial supply to tumor nodules, assess portal vein blood flow, and detect possible anatomic variants of arterial liver vessels. In addition, planar scintigraphy and single-photon emission tomography co-registered with a CT were performed following the injection of Tc-99 macroaggregated albumin (Tc99-MAA) to assess the lung-shunt fraction, visceral shunting, and preferential tumor flow.

Following general recommendations, an elevated hepatopulmonary shunt leading to exposure of the lungs of >30 Gy in a single session or the failure to prevent deposition of microspheres in extrahepatic abdominal locations were exclusion criteria for therapy with TARE. With one exception, treatment was performed sequentially, first treating the liver lobe with the major fraction of the tumor burden. The mean time between the first and second lobar TARE was 28 days (range 23 to 37 days). Activity was calculated using a single compartment model to achieve a dose of about 120 Gy. The mean dose applied was 117 Gy (range 97 to 140 Gy, one patient in each case with the minimum and maximum dose).

Patients treated systemically initially received atezolizumab 1200 mg plus bevacizumab 15 mg/kg body weight intravenously every three weeks after esophageal variceal treatment commenced, if applicable. Medication was discontinued in cases of severe adverse events or upon tumor progression. After discontinuation, patients received subsequent treatments according to current guidelines. The presence of an underlying autoimmune hepatitis was a contraindication for the combination therapy.

### 2.4. Clinical Data and Follow-Up

Clinical and biochemical data were measured at baseline, before the second TARE, and then 28 and 90 days after the second radioembolization, followed by regular follow-ups every three months. MRI or CT scan was performed at baseline, 90 days after the second TARE, and every three months thereafter concomitantly with clinical follow-up. The therapy response assessment was based on clinical and radiological evaluation according to HCC-specific modified RECIST (mRECIST) criteria. Treatment-related adverse events were classified according to the NCI Common Terminology Criteria for Adverse Events (CTCAE) v5.0.

### 2.5. Statistical Analysis

R (version 4.3.0, R Foundation for Statistical Computing, Vienna, Austria) was used for statistical analysis. Data are presented as mean values with standard deviation for continuous variables with normal distribution and as a median for continuous variables with non-normal distribution. For comparison between groups, univariate analysis was performed using Student’s *t*-test, the Mann–Whitney U test, or Mood’s median test as appropriate for continuous variables and the chi-square test or Fisher’s exact test for categorical variables.

Survival time was measured from the date of the first TARE treatment or application of atezolizumab/bevacizumab, respectively. For hepatic decompensation, the exact event time could not be measured in most cases, so the time was estimated by midpoint imputation for the time interval in which decompensation occurred.

Median overall survival (mOS) was calculated using the Kaplan–Meier method. Patients who were lost to follow-up were censored. Subgroups were compared using the log-rank test. Multivariate analysis and hazard ratio (HR) calculations were performed using Cox regression. All reported *p* values are two-sided and considered significant if <0.05.

## 3. Results

### 3.1. Baseline Characteristics

Seventy-four patients were included in this study (33 received bilobar TARE; 41 received ST) between January 2019 and October 2021. The reverse Kaplan–Meier estimate of median follow-up was 25.4 months (95%CI: 13.7 months–∞) for the TARE group and 19.5 months (95%CI: 14.1 months–29.7 months) for the group receiving ST. The difference can be attributed to the fact that atezolizumab/bevacizumab was only applied after data from the IMBrave150 trial had been published in early 2020. Patient characteristics for the groups undergoing radioembolization and systemic therapy, respectively, are summarized in Table 1.

The composition of the two groups regarding demographic criteria (age, gender) and liver function (assessed using both Child–Pugh and ALBI scores) did not differ significantly. Most patients were male (71.6%), and the mean age at the beginning of treatment was 69.5 years (SD 10.6 years). Fifty-four patients had been diagnosed with liver cirrhosis, most of whom had compensated liver function (CPS A, ALBI 1) at baseline. However, both groups differed significantly concerning the presence of extrahepatic tumor manifestations. TARE was performed in patients without extrahepatic spread, whereas systemic therapy was administered in cases of advanced HCC (extrahepatic spread or intrahepatic tumor burden >50%). Only one patient with a BCLC A tumor stage received systemic therapy because of previous acute-on-chronic liver failure and concurrent oral floor carcinoma treated with external beam radiation. Two other patients in the ST group also suffered from other malignancies: one from squamous cell carcinoma of the lung, which was resected, and one from urothelial carcinoma, which remained stable under therapy with atezolizumab/bevacizumab.

In the TARE group, six patients (18.2%) received subsequent therapies upon tumor progression, and four patients (12.1%) received systemic therapy with lenvatinib or sorafenib following radioembolization. Two patients (6.1%) were subsequently treated locally with DSM-TACE (trans-arterial chemoembolization with degradable starch microspheres) using EmboCept S microspheres (PharmaCept GmbH, Berlin, Germany). In the ST group, nine patients (22.0%) received subsequent therapy with sorafenib or lenvatinib, and one patient (2.4%) received nivolumab/ipilimumab. Two patients (4.8%) were treated with bilobar TARE upon tumor progression, and one (2.4%) was referred to surgery for metastasectomy.

### 3.2. Efficacy

**Figure 1:**
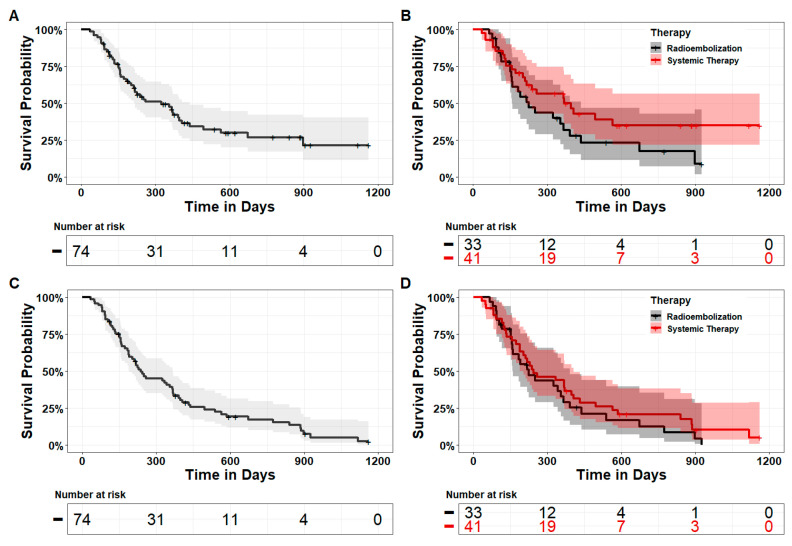
Plots of Kaplan–Meier survival estimates with corresponding 95% confidence intervals. (**A**) Overall survival for the entire cohort (mOS 324 days). (**B**) Overall survival for TARE vs. ST (mOS TARE 225 days; mOS ST 370 days, *p* = 0.12). (**C**) Progression-free survival for the entire cohort (mPFS 173 days). (**D**) Progression-free survival for TARE vs. ST (mPFS TARE 184 days; mPFS ST 173 days, *p* = 0.6).

Although there was a trend to longer median overall survival in the ST group compared to the TARE group [12.1 months (95%CI 7.1 months–∞) vs. 7.4 months (95%CI interval 5.2 months–14.4 months)], the difference was not statistically significant (*p* = 0.12) (Figure 1A,B). No statistical difference could be found in overall survival when comparing subgroups with cirrhosis (HR 0.74, 95%CI: 0.39–1.41, *p* = 0.4), BCLC B tumor stage (HR 0.79, 95%CI: 0.17–3.60, *p* = 0.8), and tumor burden > 25% (HR 0.91, 95%CI: 0.34–2.43, *p* = 0.8).

Regarding tumor response, significantly more patients reached at least a partial response in the TARE group than in the ST group (42.4% vs. 14.6%, *p* = 0.009). However, the disease control rate was similar in both groups (45.5% in the TARE group and 58.5% in the ST group, *p* = 0.35), perhaps explaining why the difference in overall response rate did not translate into a survival benefit.

Median progression-free survival (PFS) was 5.7 months in the TARE group (95%CI: 4.4–7.1 months) and 6.0 months in the ST group (95%CI: 5.0–8.9 months) without a statistical difference between both groups (*p* = 0.6) (Figure 1C,D). When only considering patients in the TARE group who did not show hepatic decompensation, the median PFS was 8.9 months (95%CI: 4.4 months–∞), while it was 3.6 months (95%CI: 2.6 months–∞) in patients receiving ST who did not have extrahepatic tumor spread.

### 3.3. Safety

In the TARE group, 20 patients (60.6%) experienced REILD shortly after TARE. It manifested as hepatic decompensation clinically and in laboratory analysis (increase of Child–Pugh score of 2 or more compared to baseline) with clinical signs of malaise. Tumor progression, biliary obstruction, infections, and other causes of acute hepatitis were ruled out in all these cases. The median time to hepatic decompensation was 106 days (95%CI 64–∞) or 75.6 days after radioembolization of the second liver lobe. One patient died shortly after TARE due to severe pancreatitis, where the association with treatment could not be ruled out. The remaining patients exhibited stable liver function clinically and in biochemistry (no change in Child–Pugh class or ALBI grade) until censoring or until a subsequent therapy was started.

**Figure 2:**
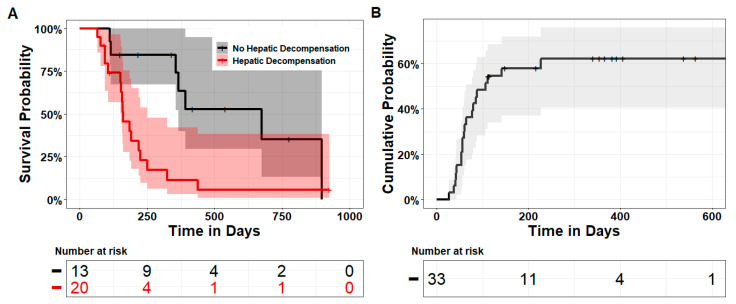
Plots of Kaplan–Meier estimates for overall survival and cumulative incidence of hepatic decompensation after TARE. (**A**) Overall survival in the TARE cohort as stratified according to the occurrence of hepatic decompensation. (**B**) Plot of the estimate for cumulative risk of hepatic decompensation. The median time to hepatic decompensation was 106 days (95%CI 64–∞).

Cox proportional hazards regression with time-dependent covariates showed that death in the TARE group was significantly associated with hepatic decompensation with a HR of 3.5 (95%CI 1.4–8.6, *p* = 0.008). As could be expected, the median overall survival was significantly shorter in patients who exhibited REILD [5.2 months (95%CI 5.0–8.2) vs. 22.1 months (95%CI 12.0–∞), *p* = 0.005] (Figure 2).

Notably, only patients with liver cirrhosis experienced REILD (*p* < 0.001). Liver function at baseline was a predictor for REILD with a hazard ratio of 3.3 [95%CI 1.05–10.4, *p* = 0.041] for patients with an ALBI grade 2 at baseline and a hazard ratio of 7.0 [95%CI 1.37–35.5, *p* = 0.019]. Liver function as stratified according to the Child–Pugh Score, however, did not predict the occurrence of REILD.

The number of severe adverse events (CTCAE grade 3 or higher) in the ST group was 15 (36.6%) in total, which is significantly lower than in the TARE group (*p* = 0.02). Among them were five (12.2%) grade 5 AEs, two (4.9%) due to bleeding complications from variceal bleeding, and one (2.4%) due to bleeding from peptic ulcer. One patient suffered from myopathy with dropped head syndrome, and one from acute TIPS (transjugular intrahepatic portosystemic shunt) thrombosis. In two cases (4.9%), the gastrointestinal bleeding complication was non-fatal but required hospitalization and led to the discontinuation of atezolizumab and bevacizumab despite partial and complete therapy response, respectively.

Hepatic decompensation occurred in five patients (12.2%) in the ST group, two (4.9%) showing impaired liver function under therapy with atezolizumab/bevacizumab and one under subsequent therapy with lenvatinib. In this context, decompensation was defined as new clinically overt hepatic complications such as ascites, hepatic encephalopathy, or variceal bleeding, regardless of whether the tumor burden had increased. Spontaneous bacterial peritonitis was detected in one patient developing new-onset ascites under therapy with atezolizumab/bevacizumab. Two other patients experienced hepatic decompensation after surgery not related to hepatocellular carcinoma. In total, during treatment, liver function deteriorated in twenty patients (48.8%), with an increase in CPS of one in seventeen patients (41.5%) and of two in two patients (4.9%). The patient who developed spontaneous bacterial peritonitis was the only one, with an increase in CPS of three (2.4%).

Two patients who showed stable disease in multiple CT scans under therapy with atezolizumab/bevacizumab exhibited significant weight loss of more than 20% of body weight over ten months, which eventually proved fatal.

## 4. Discussion

Recent advances in the systemic treatment landscape include the introduction of immune checkpoint inhibitors, which represent a more efficacious systemic therapy, improving overall survival while being more tolerable than traditional tyrosine- and multi-kinase inhibitors. Compared to sorafenib, better tolerability has also been reported for TARE with fewer adverse events and a higher quality of life [11,12]. In fact, recently, an indirect comparison between patients in the SARAH and IMbrave150 trial has shown similar time to deterioration of quality of life [18]. With reports showing a similar safety profile, we aimed to identify patients who would benefit from TARE or atezolizumab/bevacizumab and improve individual treatment allocation. This study compares the safety and efficacy of unselective bilobar TARE and atezolizumab/bevacizumab as first-line treatment options for locally advanced unresectable HCC.

In TARE, the antitumoral effect is due to radiation rather than embolization, and tumor response and liver toxicity due to radiation affecting non-tumorous tissue are dose-dependent [19,20]. Therefore, much research in recent years has focused on personalized dosimetry approaches. The traditional dosimetric approach used in patients in this study prescribes a dose of 120 Gy to the perfused volume of treated liver tissue to balance the desire to achieve a tumoricidal dose against the safety of necessarily treating nontarget hepatic parenchyma. It is based on a “single compartment” model, which assumes that the radiation dose delivered by the Y90 microspheres is distributed uniformly throughout the entire volume of the treated tissue. However, this method does not entirely reflect the reality of the situation because the hypervascularization of the tumor leads to a higher deposition of Y-90 microspheres in the tumorous tissue. To account for this, the currently favored dosimetric approach utilizes compartment modeling, in which the predicted doses to the tumor and non-tumor liver parenchyma (based on prior Tc99-MAA distribution) are assessed separately to achieve sufficient tumor doses while not exceeding safety thresholds in the non-tumor liver tissue. Garin et al. introduced personalized dosimetry when they showed that tumor doses above 205 Gy were predictive of tumor response and overall survival [21]. This threshold was confirmed in subsequent studies [22,23], leading to the DOSISPHERE-01 trial, demonstrating a higher median overall survival in the personalized dosimetry group compared to the standard dosimetry group while maintaining an acceptable safety profile [24]. However, this trial included patients with mostly unifocal and unilobar HCC, such that at least 30% of healthy livers remained radiation-free. In our study, we focused solely on patients with locally advanced HCC with multifocal, bilobar disease such that palliative radioembolization required unselective treatment of the whole liver. Under these circumstances, 60.6% of patients experienced REILD following TARE, supporting the theory that hepatic decompensation depends on the volume of the irradiated liver. This result is significant because the occurrence of REILD was shown to negatively impact survival: Overall survival was significantly higher in TARE patients who did not exhibit hepatic decompensation. However, the percentage of patients experiencing REILD in our study was substantially higher than that reported in other studies [11,13,15,20,24].

On the one hand, significant variations may be explained by the small patient populations. On the other hand, different application forms of TARE (glass vs. resin microspheres) may also play a role. Lastly, we focused on patients with multifocal, bilobar disease, suggesting that most of our patients had further advanced disease stages than in the studies cited above.

There is also a discrepancy in overall survival in our patients receiving ST and the mOS of 19.2 months reported in the IMbrave150 study cohort [25]. This difference in survival could be accounted for by our patients’ comorbidities and HCC etiology. In our study, we also treated patients with impaired liver function (ALBI grade 3) and patients beyond the IMbrave150 inclusion criteria, e.g., patients with significant comorbidities such as cardiovascular disease and other malignancies, who are known to have less of a survival benefit from treatment with atezolizumab plus bevacizumab [16]. Another factor to consider is the etiology of HCC in our cohort, as most of our patients in the ST group had HCC of non-viral origin. Subgroup analysis of the IMbrave150 study as well as a multi-center retrospective analysis suggested that atezolizumab/bevacizumab prolong overall survival over TKIs only in HCC patients of viral etiology [25,26]. Even though patients generally received sequential therapies with TKIs such as lenvatinib or sorafenib, deteriorating liver function during initial therapy adversely affects the efficacy of subsequent treatment, emphasizing the significance of the choice of first-line treatment. Despite these factors limiting overall survival in the ST group and the fact that these patients had a higher tumor burden than in the TARE group or already exhibited extrahepatic tumor manifestations, the high percentage of REILD occurrence in the TARE group led to a similar overall survival in both groups.

In line with other studies, we identified liver cirrhosis as the main predictor of hepatic decompensation [15]. In our cohort, no patient without liver cirrhosis developed REILD. In patients with liver cirrhosis, even the suggested dose reduction of 10–20% did not result in a better outcome, as even a dose of 97 Gy, corresponding to a 19.2% dose reduction, still led to hepatic decompensation in our study. The significant prognostic factor for the occurrence of REILD in cirrhotic patients was their liver function at baseline. Notably, while the ALBI score was able to predict the prognosis of patients after TARE—even patients with an ALBI grade 2 had a threefold increase in the risk of developing REILD—the CPS was not. Our results are in line with other reports which link the occurrence of REILD to the pretreatment bilirubin level [27]. In particular, the ALBI score has been shown to provide an improved prognostic stratification capability over CPS regarding survival after radioembolization [28,29] and to predict REILD more accurately [30]. Altogether, our results underscore previous observations that radiation toxicity in TARE is a function of both liver function and the volume of the liver treated [31].

Our study has several limitations. First, due to the retrospective nature of this study, an intention-to-treat analysis could not be executed, even though patients were, in fact, followed prospectively. Additionally, our study constitutes a single-center analysis with a restricted patient cohort. Moreover, it is possible that survival within the TARE group is underestimated, given the substantial disparity in time between the treatment decision and the actual administration of treatment, particularly compared to the ST group. This difference is owed to the fact that prior angiography and MAA-scintigraphy had to be planned for TARE. Despite these limitations, this study showed that careful patient selection is crucial and that REILD poses a significant risk in whole-liver treatment of cirrhotic patients, especially with an increased ALBI grade (ALBI grade ≥ 2) at baseline.

Recent years have seen remarkable advances in the systemic treatment landscape of HCC. Several combination therapies with ICI have been shown to outperform traditional tyrosine- and multi-kinase inhibitors like sorafenib and lenvatinib [32,33,34], which has led to a prioritization of systemic treatment for patients with intermediate-stage HCC (BCLC B) with diffuse, extensive bilobar liver involvement in the latest update of the BCLC treatment strategy 2022 [3]. Several recently updated publications and guidelines [35,36] consider the combination of atezolizumab and bevacizumab as the standard of care for first-line therapy of unresectable HCC patients. However, the European Medicines Agency (EMA) approved the treatment only in late 2020, so it was not widely available at the time of treatment of our patients who underwent bilobar TARE. At that time, petitioning for the application of atezolizumab-bevacizumab at health insurance companies meant a delay of treatment of several weeks to months, which is a significant amount of time considering the short life expectancy without treatment in advanced-stage HCC. Time was especially a concern in cases where patients suffered from multifocal or bilobar HCC, in whom we aimed to achieve a significant reduction in tumor mass over a short period, as we hoped it would translate into enhanced survival. Our data confirm that a higher overall response rate was achieved with TARE than with ST, even though this did not translate into longer overall survival, given that the disease control rate was similar in both groups.

As systemic treatment has been increasingly recommended for earlier BCLC tumor stages, the role of TARE has evolved as a potentially curative treatment for early-stage HCC (BCLC A). Ablative radioembolization, also known as radiation segmentectomy, refers to the selective administration of high-radiation doses to one or two liver segments to achieve necrosis of the entire angiosome treated [37,38]. Among others, the LEGACY study supports the notion of TARE as a standalone treatment for early-stage disease when current guideline recommendations such as resection or thermal ablation are not feasible [39].

Despite the risk of REILD, bilobar TARE remains a treatment alternative in patients without cirrhosis. HCC without cirrhosis is expected to become increasingly common due to the rising incidence of nonalcoholic fatty liver disease, a precancerous condition that may lead to HCC without cirrhosis. Although immunotherapy shows promising results, efficacy is inconsistent and individual response to treatment is unpredictable. Especially in HCC patients without cirrhosis who show progression under therapy with atezolizumab/bevacizumab, TARE might be considered to achieve a durable response. Furthermore, bilobar TARE remains a treatment option for patients with contraindications for immunotherapy, such as autoimmune hepatitis or other severe autoimmune conditions, as well as patients with HCC recurrence after liver transplantation [40].

Given these developments in TARE and systemic treatment, an intriguing concept currently being investigated is the combination of TARE with ICI. There is growing evidence that radiation might induce an immune response by releasing tumor-associated antigens into circulation and facilitating tumor recognition by CD8-positive T cells [41,42]. This suggests that combination therapies with TARE and ICI might be an alternative to whole-liver TARE, and several trials are currently ongoing.

## 5. Conclusions

Depending on the selectivity of application and the dose applied, the utility of radioembolization ranges from curative treatment in early-stage hepatocellular carcinoma to palliative treatment in advanced-stage disease. If used for the latter, whole-liver radioembolization can be an effective treatment suitable for achieving a durable tumor response in a select group of patients. However, patients with diagnosed liver cirrhosis have a significant risk of developing severe radioembolization-induced liver disease, limiting its applicability for treatment. This risk increases with declining liver function as measured by the ALBI grade. When liver function is impaired (ALBI grade ≥ 2), the treatment stage migration strategy suggests moving from local to systemic therapy to prevent REILD. Careful patient selection and personalized dosimetry could help improve the outcome and lower toxicity.

## Figures and Tables

**Table 1:** Baseline characteristics of all included patients.

	All Patients (n = 74)	Patients Undergoing Radioembolization (n = 33)	Patients Receiving Systemic Therapy (n = 41)	*p*-Value (RE vs. ST)
Age (years), mean ± SD	69.5 (10.6)	71.1 (9.7)	68.1 (11.2)	0.23
Male gender, N (%)	53 (71.6)	22 (66.6)	31 (75.6)	0.44
ECOG Performance Score				
0, N (%)	20 (27.0)	6 (18.1)	14 (34.1)	0.32
1, N (%)	49 (66.2)	24 (72.7)	25 (61.0)	
2, N (%)	5 (6.8)	3 (9.1)	2 (4.9)	
Liver Cirrhosis, N (%)	54 (73.0)	27 (81.8)	27 (65.9)	0.19
Underlying Liver Disease				
ASH, N (%)	17 (23.0)	7 (21.2)	10 (24.4)	0.45
NASH, N (%)	23 (31.1)	13 (39.4)	10 (24.4)	
HCV, N (%)	16 (21.6)	5 (15.2)	11 (26.8)	
HBV, N (%)	3 (4.1)	2 (6.1)	1 (2.4)	
Autoimmune, N (%)	2 (2.7)	2 (6.0)	0 (0)	
Other, N (%)	11 (14.9)	3 (9.1)	8 (19.5)	
Child–Pugh Score				
A, N (%)	67 (90.5)	32 (97.0)	35 (85.4)	0.12
B, N (%)	7 (9.5)	1 (3.0)	6 (14.6)	
C, N (%)	0 (0)	0 (0)	0 (0)	
ALBI grade				
1, N (%)	54 (73.0)	27 (81.8)	27 (65.9)	0.11
2, N (%)	8 (10.8)	4 (12.1)	4 (9.8)	
3, N (%)	12 (16.2)	2 (6.1)	10 (24.4)	
Complications of Cirrhosis				
Ascites, N (%)	13 (17.6)	4 (12.1)	9 (22.0)	0.36
Refractory ascites, N (%)	3 (4.1)	0 (0)	3 (7.3)	0.25
Tumor Stage				
BCLC A, N (%)	2 (2.7)	2 (6.1)	0 (0)	0.34
BCLC B, N (%)	11 (14.9)	5 (15.2)	6 (14.6)	
BCLC C, N (%)	61 (82.4)	26 (78.8)	35 (85.4)	
Presence of macrovascular invasion, extrahepatic spread, or both, N (%)	36 (48.6)	6 (18.2)	30 (73.2)	<0.001
Extrahepatic Spread, N (%)	29 (39.2)	0 (0)	29 (70.7)	<0.001
Macrovascular Invasion, N (%)	18 (24.3)	6 (18.2)	12 (29.3)	0.29
Tumor burden > 25%, N (%)	22 (29.7)	10 (30.3)	12 (29.3)	1.0
AFP (IU/mL), median (min–max)	42.1 (1.1–256,801.9)	12.9 (1.1–2374.2)	195.4 (1.2–256,801.9)	0.16
Prior therapy				
Resection, N(%)	16 (21.6)	3 (9.1)	13 (31.7)	0.024
TACE, N(%)	17 (23.0)	11 (33.3)	6 (14.6)	0.09
RE, N(%)	8 (10.8)	2 (6.1)	6 (14.6)	0.29
Metachronous tumors, N (%)	3 (4.1)	0 (0)	3 (7.3)	0.25

ECOG PS: Eastern Cooperative Oncology Group performance status, ASH: alcoholic steatohepatitis, NASH: nonalcoholic steatohepatitis, HCV: hepatitis C virus, HBV: hepatitis B virus, CPS: Child-Pugh score, ALBI grade: albumin-bilirubin grade, ALBI score: albumin-bilirubin score, BCLC: Barcelona Clinic Liver Cancer, TACE: transcatheter arterial chemoembolization, TARE: transarterial radioembolization.

## Data Availability

Data analyzed in this study are available from the corresponding author on reasonable request.
